# Supplementary material for: Real world external validation of metabolic gestational age assessment in Kenya
Source: PLOS Glob Public Health. 2022 Nov 28;2(11):e0000652. doi: 10.1371/journal.pgph.0000652 (PMC10021775; doi:10.1371/journal.pgph.0000652)
Supplement: S1 Appendix — (DOCX) [file pgph.0000652.s001.docx]

**Real world external validation of metabolic gestational age assessment in Kenya**

**S1 Appendix: Supplementary Methods**

**Data preparation**

In preparation for use in modeling, newborn screening analytes were winsorized using an adapted “Tukey Fence” approach [1]. For each analyte, this involves assigning values more than three interquartile ranges above the third quartile (the upper Tukey fence) or below the first quartile (the lower Tukey fence), to the Tukey fence value, or the smallest/largest observed value in the dataset, whichever was the least extreme. This approach preserves much of the “extremeness” of outliers but prevents extreme values from disproportionately impacting model building and parameter estimation. The majority of measured analytes exhibit strongly right-skewed distributions, which was addressed though natural log transformation, which also stabilizes the variance, reducing the impact of heteroskedasticity. Finally, both analyte levels and birth weight values were normalized by subtracting the mean and dividing by the square root of the standard deviation for each variable (pareto scaling) [2,3], which centers all predictors to have a mean of zero, and scales them to reduce the impact of variations in dispersion of individual analytes across cohorts.

**Model development in the Ontario, Canada model derivation cohort**

Briefly, a cohort of 159,131 infants born between January 2012 and December 2014 was derived from the BORN Ontario birth registry which included all required predictors as well as reference standard GA measured by confirmed 1^st^ trimester gestational dating ultrasound.

For Models 1 and 2, birth weight was modeled using a restricted cubic spline with five knots to allow for non-linearity of the association of birth weight with gestational age (GA). For Model 2, we included all pre-specified covariate main effects in the models, however we additionally identified the most predictive analytes using the metric of generalized partial Spearman correlation that detects non-linear and non-monotonic associations with GA, mutually adjusted for all other analytes and clinical covariates. Based on this partial Spearman correlation analysis there were seven analyte covariates that had distinctly stronger partial correlations with GA compared to all others. These seven analytes were modeled using restricted cubic splines with 5 knots. These were (in order of strength of partial spearman correlation): fetal-to-adult hemoglobin ratio, 17-OHP, C4DC, TYR, ALA, C5, and C5DC. For birthweight, and the seven strongest analyte predictors, knot placement was at the 5th, 27.5th, 50th, 72.5th, and 95th percentiles based on the Ontario population distribution of these analytes [4].

For Model 1, all covariates and pairwise interactions were included in the model without variable selection or regularization. For Model 2 we employed Elastic Net regularization, which employs two forms of penalization (called L1 and L2 regularization) to simultaneously estimate regression coefficients while also shrinking them towards zero to penalize the increase in model complexity from each additional term included in the model [5]. The Elastic Net regression methodology allows models to be fit with a large number of predictors, even models where the number of predictors exceeds the number of observations (p>>n), and provides strong protection against overfitting, and against the instability inherent in fitting models with a large number of predictors relative to the number of observations available for model fitting [5,6].

Given the strong imbalance in preterm gestation versus term and post-term gestation in our population cohort of infants, we used a weighting scheme to reduce the dominance of term infants in model development. We used an ad hoc approach to developing the weighting scheme in which preterm and post-term infants were upweighted in model training. We did not strictly derive the weights to give equal weight to all GAs, as when we did this, overall model performance was negatively impacted. We iteratively increased the weights in preterm and post-term infants until we identified a threshold where the internal validation MSE in preterm/post-term infants was maximized but MSE in term infants hadn’t declined substantially. These weights were developed in earlier model building studies, and the same weighting scheme was used in the current study. Hence development of weights was not undertaken in any of the model training, validation or testing data from the current study. The weights used were as follows:

22-33 weeks: weight=10

34-35 weeks: weight=5

36-37 weeks: weight=2

38-40 weeks: weight=1

41+ weeks: weight=2

**Bootstrap confidence intervals**

Given the fact that parametric standard error estimates were not readily calculable for our validation performance metrics, valid parametric confidence intervals could not be calculated. We therefore calculated bootstrap percentile confidence intervals based on the 2.5th and 97.5th percentiles of performance metrics from 1000 bootstrap samples.

**Validation of GA estimation models**

Our external validation protocol has been reported elsewhere [7]. We applied the identical approach in validating models internally (in Ontario test cohort) and externally in the Kenya cohort.

Final Ontario regression model equations were used to calculate an estimated GA in the Ontario test cohort and in the Kenya cohort. For each infant, model performance was assessed by comparing the estimated GA from the model to the ultrasound-derived GA and calculating the difference between the model estimate and ultrasound-based estimate. Agreement was measured by calculating the mean absolute error (MAE) measured in weeks (the average of the absolute values of the model vs. ultrasound difference across all observations). Lower MAE reflects higher accuracy. We also calculated the percentage of infants with GAs correctly estimated within 7 and 14 days of ultrasound-based GA. We assessed model performance overall and in important subgroups: preterm birth (<37 weeks gestation), and small-for-gestational age (SGA10). SGA10 was defined as birthweight below the 10^th^ percentile within categories of gestational week at delivery and infant sex, respectively. The percentiles were calculated based on INTERGROWTH-21 sex and GA categories [8].

All reported performance metrics were reported as the mean, and 2.5^th^ and 97.5^th^ bootstrap confidence limits.

**References**

1. Tukey JW. Exploratory data analysis. 1st ed. Reading: Addison‐Wesley Publishing Company; 1977.

2. van den Berg RA, Hoefsloot HCJ, Westerhuis JA, Smilde AK, van der Werf MJ. Centering, scaling, and transformations: Improving the biological information content of metabolomics data. BMC Genomics. 2006;7. doi:10.1186/1471-2164-7-142

3. Eriksson L, Johansson E, Kettapeh-Wold S, Wold S. Scaling. Introduction to multi- and megavariate data analysis using projection methods (PCA & PLS). Umetrics; 1999. pp. 213–225.

4. Harrell FE. Regression Modeling Strategies with Applications to Linear Models, Logistic and Ordinal Regression and Survival Analysis. 2nd ed. New York: Springer;

5. Zou H, Hastie T. Regularization and variable selection via the elastic net. J R Stat Soc Ser B Stat Methodol. 2005. doi:10.1111/j.1467-9868.2005.00503.x

6. Hastie T, Tibshirani R, Friedman J. The Elements of Statistical Learning : Data Mining, Inference, and Prediction. 2nd ed. New York: Springer; 2008.

7. Murphy MSQ, Hawken S, Atkinson KM, Milburn J, Pervin J, Gravett C, et al. Postnatal gestational age estimation using newborn screening blood spots: a proposed validation protocol. BMJ Glob Health. 2017;2: e000365. doi:10.1136/bmjgh-2017-000365

8. Villar J, Giuliani F, Fenton TR, Ohuma EO, Ismail LC, Kennedy SH. INTERGROWTH-21st very preterm size at birth reference charts. The Lancet. Lancet Publishing Group; 2016. pp. 844–845. doi:10.1016/S0140-6736(16)00384-6
