# Supplementary material for: Real world external validation of metabolic gestational age assessment in Kenya
Source: PLOS Glob Public Health. 2022 Nov 28;2(11):e0000652. doi: 10.1371/journal.pgph.0000652 (PMC10021775; doi:10.1371/journal.pgph.0000652)
Supplement: Attachment — Submitted filename: ResponsetoReviewers_Final.docx [file pgph.0000652.s002.docx]

PGPH-D-22-00803
Real world external validation of metabolic gestational age assessment in Kenya
PLOS Global Public Health

**Journal Requirements:**

1. Please update your online Competing Interests statement. If you have no competing interests to declare, please state: “The authors have declared that no competing interests exist.”

**Author response:**

**We have updated our competing interest statement.**

2. We have noticed that you have uploaded Supporting Information files, but you have not included a list of legends. Please add a full list of legends for your Supporting Information files after the references list.

**Author response:**

**We have added the full list of legends for the Supporting files as requested.**

3. Please review your reference list to ensure that it is complete and correct. If you have cited papers that have been retracted, please include the rationale for doing so in the manuscript text, or remove these references and replace them with relevant current references. Any changes to the reference list should be mentioned in the rebuttal letter that accompanies your revised manuscript. If you need to cite a retracted article, indicate the article’s retracted status in the References list and also include a citation and full reference for the retraction notice.

**Author response:**

**We confirm that the reference list is complete and correct.**

**Reviewers' comments:**

**Reviewer #1:** This manuscript is addressing a fundamental problem in maternal-child global health which is how can the gestational age (GA) at birth of an infant be determined in a low resource setting where a gold standard of first trimester US is impractical. Fundal height, LMP, Ballard like scores all have large inaccuracies, especially in the face of infants being SGA but recently biochemical phenotyping of the mother and/or fetus have shown promise. This group and others have previously shown the data from newborn screening can provide an accurate estimate and this provides a reasonable alternative if the data is needed to determine the population prevalence of preterm birth, the range of gestational ages for newborns or whether a new intervention is changing the gestational age at delivery. In this study the investigators look to see if methods and algorithms developed in high resource settings can be used reliably in an LMIC setting (Kenya in this case). The report adds important data to the struggle to find accurate and effective tools to determine GA in LMICs. Overall the data is well collected and presented and the report well analyzed and written and appropriately cautious in its conclusions. There are a few concerns.

The last sentence of the abstract could be written with more specificity and impact. We have known for at least 3 decades that early US provides the best estimates of GA and any future studies in LMICs will likely use that information in a research setting, as they have done, with a study design that would target early US as the gold standard. Rather the conclusion should be what this study specifically shows.

**Author response:**

**We have modified the last paragraph of the Abstract as follows:**

**“The accuracy of metabolic model-based GA estimates in the Kenya cohort was lower compared to our previously published internal validation studies. However, inconsistency in the timing of reference dating ultrasounds appears to have been a contributing factor to diminished model performance, highlighting the challenges in reliably estimating GA in low resource settings.”**

***************************************************************************
While the authors rightly point out that “medical needs and developmental milestones differ between term, preterm and SGA infants and thus, accurately identifying at-risk infants at birth is important in informing their postnatal care” the use of metabolic dating done after birth will have limited utility for the medical management of newborns as the results will come in a couple of days at best after testing when the most critical needs will have been addressed by the clinical state of the infant. It would seem that most utility of such testing would be to determine population frequencies of GA or preterm birth rates and/or to determine if improvements in PTB or SGA rates have occurred after interventional studies addressing these needs are underway. They do a better job of this in the discussion.

**Author response:**

**We agree with the reviewer, and have edited the first paragraph of the introduction to hopefully make this distinction clearer:**

“Furthermore, medical needs and developmental milestones differ between term, preterm and SGA infants. Thus, accurately identifying at-risk infants at birth is important, both for estimating the true population burden of preterm birth, and potentially in informing postnatal care and supportive resources.”

***************************************************************************

Nonetheless it’s a bit confusing as to whether the conclusion here is that we need a higher percentage of early US or that metabolic screening works. Its not clear why they could not get more compliance around appropriately timed US given that they describe this as a prospective pregnancy cohort. There are many other LMIC studies in which surveillance is done for timing of pregnancy at two or even one month intervals where detection by missed LMP can then lead to US at an optimal time.

**Author response:**

**There is evidence that performance was better when we looked at US done in the optimal window. This study didn’t necessarily support that the metabolic screen works in this setting, but that one factor contributing to the apparent poorer performance of the model could be the inconsistency / heterogeneity in early US timing. Our study was designed as an implementation study, rather than a highly controlled cohort study. This highlights some of the challenges in implementing and evaluating interventions such as ours in real world LMIC settings as opposed to controlled trials/cohort studies.**

***************************************************************************
I am unable to address issues related to the references cited in that the version of the manuscript I had to review (sent twice) were missing most of the references that should have been on pages 8 and 9. Appropriate citations will need to be assessed prior to acceptance and publication.

**Author response:**

**Apologies for the issues with the references, we have resolved this and have presented the correct list of references.**

In the Bota methods reference paper it refers to plans to analyze based on 3 models but only 2 are reported here. Why the change?

**Author response:**

**We have previously evaluated a third model which included sex, multiple gestation and analytes (excluding birthweight). In all of our previous validations, models excluding birthweight performed more poorly than models including birthweight. Since birthweight is essentially universally available, we have only presented findings for metabolic models including birthweight, and one using only sex, multiple gestation and birthweight as a baseline comparison to illustrate the predictive value added by the metabolic markers.**

***************************************************************************

Why do they include multiple births at all in this study and then adjust for them? Its well known the twins and above deliver earlier than singletons and this seems an unnecessary confounder for a study of this type.

**Author response:**

**We made the early decision to develop a model that was as broadly applicable as possible, hence we did not exclude multiple gestations, but rather included it as a predictor in the model. We agree that this might not be useful clinically, as multiples will always receive an elevated level of care, and are very often born preterm, but we found that overall model performance was not impacted negatively by including multiples in the model, and that the models do perform well in multiples, though not as well as in singletons.**

***************************************************************************
They appear to have switched from MRE to MAE as a primary metric in reporting. Is there a reason for that?

**Author response:**

**Both RMSE and MAE are in the same units as the outcome (GA in weeks), and both broadly estimate the average accuracy of model predictions vs. Reference GA (1st trimester ultrasound dating). However, because RMSE is based on squared residuals, it tends to give more weight to larger residuals. Because MAE is based on absolute values of residuals (ie. removing the sign) it doesn’t have the property of inflating the influence of larger residuals. Therefore, a growing number of authors, ourselves included, feel that MAE is an informative metric of “average precision” that is less sensitive to outliers. We continue to include RMSE in order to facilitate comparisons of our model performance to the work of others, as well as our own previous publications. We have added a statement to this effect in the last paragraph of the Methods section.**

**“MAE is less sensitive to outliers (large residuals) and is a helpful metric of “average error” which is often reported in model validation studies with continuous outcomes. We have included both metrics to facilitate comparisons with other study results.”**

***************************************************************************

In the supplement they refer to 159,000 infants being used in model development from Ontario but in the primary table its 39,000. What is the difference?

**Author response:**

**The Ontario results presented are for the Ontario testing cohort (N=39K) which was set aside for internal validation, whereas the model was developed in the model development cohort (N=159K).**

***************************************************************************
Minor Comments:
In the Bota, Gates Open Research paper the PTB rate in Kenya was pegged at 19%. They now have very different results so a short discussion around this might be of interest.

Similarly the Ontario data had much lower PTB and SGA rates as they comment on. Could they also comment on what affect this might have on results (i.e. presumably given the high prevelance of GA around 39 weeks it might make the Ontario data more precise just based on the distribution being tighter.

**Author response:**

**In the Gates Open Research paper, we stated that previous studies had estimated the preterm birth rate to be 19%. As we have observed in our other validation studies, there is participation bias against very preterm and extremely preterm**

**infants, whose parents express reluctance to consent to the study. As a result, we had a relatively small number of samples collected from very preterm and**

**extremely preterm infants. Model-estimated gestational ages were most accurate in infants born close to full-term, and were overestimated in preterm infants and underestimated in post-term infants. We added the following text to the Discussion:**

**“Further, as observed in our previous validation studies, the study sample was affected by a participation bias against preterm and extremely preterm infants. Model estimated gestational ages were most accurate in infants born close to full-term, and tended to be overestimated in preterm infants and underestimated in post-term infants.”**

**We expected and did observe the model performance to be lower in preterm and SGA infants. Therefore, with a higher proportion of preterm and SGA infants in the study sample from Kenya compared to Ontario, the overall model performance is decreased, driven by lower performance in the preterm and SGA infants. This would result in overall model accuracy (MAE) appearing worse, which is why we also present MAE stratified by important subgroups such as SGA/preterm.**

***************************************************************************

They state parental consent was given. Does this mean both parents?

**Author response:**

**Consent was sought from the mothers. Some mothers consulted their spouse / partner in their decision but consent was not required from both parents. We have clarified this in the text to avoid any ambiguity.**

***************************************************************************
Fine for there to be an independent publication on the incidental findings but they might provide the numbers of positives found and which ones here.

**Author response:**

**We have added the following sentence to the end of the first paragraph of the Results section reporting the overall incidental screen positive findings.**

**“There were 11 screen positive results for hemoglobinopathies that were reported back to the study site for followup. Details of incidental screen positive findings and followup will be reported in a separate manuscript.”**

***************************************************************************
What percent of infants had their heel sample collected prior to 24 hrs and did they see any differences in the profiles from these infants?

**Author response:**

**Most heel-prick samples in the study were collected prior to 24 hours (784/1039, 75.5%). It was anticipated that many participants would be discharged within a few hours after birth, so site staff were instructed to delay collection as long as possible before mothers and babies were discharged home. We did expect differences in profiles based on age at sample collection, but we didn’t observe differences in model performance in samples collected early vs late.**

Is there a reference for there being little/no effect of storing the samples at ambient temperature (and ? humidity or are desiccants used?)

**Author response:**

**We have previously published a study comparing storage time and conditions for stored bloodspots (ref Murphy et. al. 2018, Gates Open Research. Metabolic profiles derived from residual blood spot samples: A longitudinal analysis.). The findings from that investigation suggested that storage time was the most important factor in impacting newborn screening profiles and model performance based on these profiles independently of temperature and humidity.**

***************************************************************************
Line 133 should be T-cell not t-cell.

**Author response:**

**Thanks, we have made this correction.**

***************************************************************************
Could table 1 also report the number of LGA infants.

**Author response:**

**Assuming the reviewer is referring to Table 2, we have now added LGA infants to Table 2.**

***************************************************************************
**Reviewer #2:** Overall this is nice report from an important study showing the possible use case for metabolic screening of gestational age in LMIC and reinforcing the known challenges in getting an appropriately timed US. It will inform future studies of this type and create a platform on which the true incidences of PTB and SGA can be reported and monitored with far greater accuracy.

**Author response: Thank you for your thoughtful review.**

***************************************************************************

**Reviewer #2:** This is an interesting study where the authors have tried to validate a metabolite predictison algorithm developed using data collected from a western population for estimating gestational age in a cohort at Siaya County, Kenya. The authors have used the refined version of the prediction algorithm. They have further analysed the validated models based on weeks that ultrasonography has been performed. Their model at collected a unique dataset using cutting edge methodology. The paper is generally well written and structured. However, in my opinion the paper has some limitations with regards to sample selection and reporting of results. Below I have provided few recommendations that could be improve the manuscript.


Comment 1:
To predict GA using metabolites and clinical covariates, authors have used heel prick (n=1039) and cord blood (n=1012) from the Kenya cohort. Are these samples paired? If yes, then how does the analysis behave in terms of paired samples? Is heel prick giving better RMSE than paired cord blood or vice- versa?

**Author response:**

**Almost all of the samples are paired, and we have previously published pairwise correlation results for individual analytes from paired cord and heel prick samples. We found that that paired analyte values had low to moderate correlation within newborns, with the exception of fetal and adult hemoglobin percentages for which agreement was nearly perfect. In general, our models developed in heel prick samples from Ontario always performed more poorly in cord blood samples as compared to heel prick samples. With respect to model performance metrics, we have not calculated correlation of model predictions for paired heel and cord samples within infants, but this may be interesting to investigate in future studies. We would expect it to be much higher than for individual analyte levels.**

***************************************************************************

Comment 2:
To report GA prediction using metabolites at delivery, authors have used RMSE and MAE in model-1 and 2 among heel prick and cord blood samples. So far, RMSE is a widely used variable to report GA estimation in weeks. Here the predicted GA in weeks is reported through MAE in the result section. What are the precise reasons/thoughts?

**Author response:**
**Both RMSE and MAE are in the same units as the outcome (GA in weeks), and both broadly estimate the average accuracy of model predictions vs. Reference GA (1st trimester ultrasound dating). However, because RMSE is based on squared residuals, it tends to give more weight to larger residuals. Because MAE is based on absolute values of residuals (ie. removing the sign) it doesn’t have the property of inflating the influence of larger residuals. Therefore a growing number of authors, ourselves included, feel that MAE is an informative metric of “average precision” that is less sensitive to outliers. We continue to include RMSE in order to facilitate comparisons of our model performance to the work of others, as well as our own previous publications.**
***************************************************************************

Comment 3: Have the authors observed any under or overestimation of GA or preterm rate when original prediction algorithm is tested on the Kenya cohort samples?

**Author response:**

**The preterm birth rate in the study cohort from Kenya was** **8.85% based on US, and 4.14% based on full model. Based on the heterogeneity in timing of ultrasound GA, we had lower confidence in the gold standard and didn’t present these estimates in the manuscript, however we did discuss the tendency of the models to overestimate GA in preterm infants and underestimate it in post-term infants (see response to an earlier reviewer comment above and text added to discussion):**

**“Further, as observed in our previous validation studies, the study sample was affected by a participation bias against preterm and extremely preterm infants. Model estimated gestational ages were most accurate in infants born close to full-term, and tended to be overestimated in preterm infants and underestimated in post-term infants.”**
